# Neuroticism-related personality traits are associated with posttraumatic stress after abortion: findings from a Swedish multi-center cohort study

**DOI:** 10.1186/s12905-017-0417-8

**Published:** 2017-10-02

**Authors:** Inger Wallin Lundell, Inger Sundström Poromaa, Lisa Ekselius, Susanne Georgsson, Örjan Frans, Lotti Helström, Ulf Högberg, Agneta Skoog Svanberg

**Affiliations:** 1 Department of Women’s and Children’s Health, Uppsala University, -751 85 Uppsala, SE Sweden; 2 Sophiahemmet University, Box 5605, - 114 86 Stockholm, SE Sweden; 3 Department of Neuroscience/Psychiatry, Uppsala University, -75185 Uppsala, SE Sweden; 4 Department of Clinical Science, Intervention and Technology, Karolinska Institute, -171 77 Stockholm, SE Sweden; 5 Department of Psychology, Uppsala University, Box 1225, -751 42 Uppsala, SE Sweden; 6 Department of Clinical Science and Education, Karolinska Institute, -118 83 Stockholm, SE Sweden

**Keywords:** Abortion induced, Anxiety disorders, Personality, Stress disorder, post-traumatic

## Abstract

**Background:**

Most women who choose to terminate a pregnancy cope well following an abortion, although some women experience severe psychological distress. The general interpretation in the field is that the most consistent predictor of mental disorders after induced abortion is the mental health issues that women present with prior to the abortion. We have previously demonstrated that few women develop posttraumatic stress disorder (PTSD) or posttraumatic stress symptoms (PTSS) after induced abortion. Neuroticism is one predictor of importance for PTSD, and may thus be relevant as a risk factor for the development of PTSD or PTSS after abortion. We therefore compared Neuroticism-related personality trait scores of women who developed PTSD or PTSS after abortion to those of women with no evidence of PTSD or PTSS before or after the abortion.

**Methods:**

A Swedish multi-center cohort study including six Obstetrics and Gynecology Departments, where 1294 abortion-seeking women were included. The Screen Questionnaire-Posttraumatic Stress Disorder (SQ-PTSD) was used to evaluate PTSD and PTSS. Measurements were made at the first visit and at three and six month after the abortion. The Swedish universities Scales of Personality (SSP) was used for assessment of Neuroticism-related personality traits. Multiple logistic regression analyses were performed to investigate the risk factors for development of PTSD or PTSS post abortion.

**Results:**

Women who developed PTSD or PTSS after the abortion had higher scores than the comparison group on several of the personality traits associated with Neuroticism, specifically Somatic Trait Anxiety, Psychic Trait Anxiety, Stress Susceptibility and Embitterment. Women who reported high, or very high, scores on Neuroticism had adjusted odds ratios for PTSD/PTSS development of 2.6 (CI 95% 1.2–5.6) and 2.9 (CI 95% 1.3–6.6), respectively.

**Conclusion:**

High scores on Neuroticism-related personality traits influence the risk of PTSD or PTSS post abortion. This finding supports the argument that the most consistent predictor of mental disorders after abortion is pre-existing mental health status.

## Background

An unwanted pregnancy is a concern for every affected woman. Most women who choose to terminate a pregnancy cope well and report positive feelings such as relief and release after the abortion [1, 2]. Although some women experience severe psychological distress following an abortion [3], the general interpretation in the field is that the most consistent predictor of mental disorders post abortion is the mental health issues that women present with prior to the abortion [3, 4]. However, the concern that induced abortion may cause mental health problems is often raised in the public debate [3], and although heavily criticized for methodological flaws, some researchers continue to advocate that induced abortion is associated with an increased risk for mental health problems [5, 6].

From the present study, we have previously reported that few women develop PTSD or posttraumatic stress symptoms (PTSS) after induced abortion. Risk factors for PTSD or PTSS post abortion in our cohort were young age, low educational level, nulliparity, and need for counseling before abortion [7]. In other settings, PTSD risk factors also include female sex [8–10], and type of trauma, with sexual trauma being associated with the highest risk for PTSD, followed by exposure to crime and witnessing violence [10–14]. Furthermore, pre-existing mental disorders, alcohol dependence, and problem-focused coping strategies are other factors associated with the risk of PTSD [13].

One factor that might help explain why some, but not all, traumatized people develop PTSD is the individual differences in personality traits, and predominantly the Neuroticism-related traits. Neuroticism is a stable personality trait which encompasses the tendency for a person to experience the world as threatening and distressing. Individuals with high scores on Neuroticism are typically anxious and vulnerable to stress, lack self-confidence, and are easily frustrated [15]. The trait is associated with increased risk for development of psychiatric disorders and the association between Neuroticism and depressive and anxiety disorders is well known [16–21]. Although Neuroticism and stressful life events are independent predictors of mood and anxiety disorders, they also interact such that individuals with high Neuroticism tend to experience major life events as more stressful than others do [22]. Neuroticism-related personality traits is reported to predict the PTSD response to trauma [23] and may be associated with the development of PTSD [22–25].

On the basis of previous research suggesting that Neuroticism is an important predictor for PTSD [13, 22, 23], we aimed to investigate whether high scores of Neuroticism-related personality traits is a risk factor for development of PTSD or PTSS post abortion. The study was conducted in Sweden where the abortion context differs from many other countries. According to the Swedish Abortion Act of 1974, women are guaranteed a free abortion up to the 18th gestational week. Induced abortions are only performed by physicians or specially trained nurse- midwives in public hospitals or in private clinics that have been approved by the Swedish National Board of Health and Welfare [26]. Approximately 37,000 women undergo an induced abortion every year [27]. Up to gestational week nine, women can choose the abortion method, either medical or surgical, in consultation with the gynecologist or the midwife. Women need not declare their reason for the abortion [26]. Surgical abortion is decreasing among first trimester abortions in favor of medical abortions, and today, the majority of the medical abortion before gestational week nine are performed at home [27]. If needed, women seeking abortion are also offered consultation with a social worker [26]. During the study period 79% of all abortions were performed before gestational week nine and of those 87.5% were medical abortions [28].

Thus, the primary aim in this multi-center cohort study was to compare Neuroticism-related personality trait scores of women who developed PTSD or PTSS post abortion with those of women with no posttraumatic stress prior to or after a first trimester abortion. A secondary aim was to explore the influence of violence and sexual trauma exposure for the development of PTSD or PTSS post abortion.

## Methods

This study is part of a multi-center cohort study targeting women who requested an induced abortion between September 2009 and June 2010 at the outpatient clinics of the Obstetrics and Gynecology Departments of six public hospitals in Sweden. Previous publications from the data set have addressed the prevalence of PTSD and PTSS post abortion, and the sociodemographic and clinical risk factors for PTSD or PTSS development [7, 29, 30]. All women who requested an induced abortion before the end of gestational week 12 were approached for participation, and the only exclusion criterion for the study was the inability to read and understand Swedish [29]. Women were informed about the study during their registration for the first abortion visit. Women who agreed to participate received written information about the study, together with a questionnaire (baseline assessment). They signed an informed consent and completed the first questionnaire at the clinic. Overall, 2602 women were invited, and 1514 women consented to participate. Two follow-up questionnaires were sent by post to the participating women, one at 3 months and another at 6 months after the abortion [7].

The baseline questionnaire solicited information on sociodemographic variables such as age, marital status, education, ethnicity, and tobacco and alcohol use. Supplementary information was retrieved from medical records and included parity, number of previous abortions, abortion method, place of abortion (home or at the clinic), antidepressant use, and psychosocial support during the abortion process.

### Measurements

The Screen Questionnaire-Posttraumatic Stress Disorder (SQ-PTSD) [11] was used for a self-reported diagnosis of PTSD and of PTSS at baseline, and at the 3- and 6-month assessments. The instrument is based on the Diagnostic and Statistical Manual of Mental Disorders, Fourth Edition (DSM-IV) diagnostic criteria for PTSD [31], and assesses trauma experiences as well as trauma symptoms [11]. The DSM-IV criteria are: A1) confrontation with the stressor involves actual or threatened death or serious injury, or a threat to the physical integrity of self or others; A2) responses of fear, helplessness, or horror to the confrontation; B) persistent re-experiencing of the traumatic event in intrusive thoughts, nightmares, or flashbacks; C) persistent avoidance of stimuli associated with the event and emotional numbing symptoms, described as an inability to experience any positive feelings such as love, contentment, satisfaction, and happiness; D) hyperarousal symptoms such as difficulties sleeping, concentrating, and controlling anger; E) duration of the disturbance (symptoms of criteria B, C, and D) for more than 1 month; F) disturbance causes clinically significant distress or impairment in social, occupational or other important areas of functioning [31].

In addition, exposure to violence was assessed by two questions i) Have you ever been beaten or attacked? ii) Have you ever been threatened with beating or attack? A positive response to at least one of these two questions was considered as being exposed to violence. Sexual trauma was assessed by two questions i) Have you ever been violently forced to perform sexual acts? ii) Have you ever been threatened into performing sexual acts? A positive response to at least one of these two questions was considered as being exposed to sexual trauma.

Only women who met all the DSM-IV criteria from A to F were classified as having a research diagnosis of lifetime PTSD. In previous studies different terms have been used to denote individuals who only partly meet the diagnostic criteria: sub-threshold PTSD, partial PTSD, or PTSS [32]. In the present study, the term PTSS was used, which was defined as prevalence of A1 and A2 criteria together with one or more of the re-experiencing, avoidance, or hyperarousal symptoms (B-C-D criteria).

The Swedish universities Scales of Personality (SSP) [33] instrument was used for evaluation of personality traits at the baseline assessment. SSP is a self-rating questionnaire designed to measure personality traits associated with vulnerability for psychopathology. The inventory is a revised version of the Karolinska Scales of Personality (KSP) [34]. The development of SSP improved the psychometric quality, and reduced the total number of items. Thus the SSP comprises 91 items divided into 13 scales, where previous factor analysis has yielded a three factor solution [33]. Factor 1 compromises personality scales assessing traits of *Neuroticism*; Somatic Trait Anxiety (tending to experience autonomic arousal; restlessness and tension); Psychic Trait Anxiety (worried, insecure, and anxious); Stress Susceptibility (easily stressed when hurried or facing new tasks); Lack of Assertiveness (non-assertive in social situations); Embitterment (dissatisfied, blaming, and envying others); Mistrust (suspicious, distrustful). Factor 2 includes scales assessing *Aggressiveness*; Social Desirability (socially conforming, friendly, helpful, negative loading); Trait Irritability (irritable, lacking patience); Verbal Trait Aggression (tending to express aggressive feelings in speech); and Physical Trait Aggression (tending to express aggressive feelings in action, such as getting into fights). Factor 3 includes scales assessing *Extraversion*; Impulsiveness (acting on the spur of the moment, non-planning); Adventure Seeking (needing change and action); Detachment (socially withdrawn, avoidant of involvement, negative loading) [35].

Each subscale includes seven items. The response format is a four-point rating scale in which 1 denotes “does not apply at all” and 4 denotes “applies completely”. Social Desirability and Detachment are included in the factors with reversed values. The SSP scores are transformed into *T* scores with a mean of 50 and a standard deviation of 10, based on a normative Swedish sex-stratified non-patient sample [33].

### Statistical analyses

For descriptive purposes, sociodemographic, clinical variables and personality trait scores were compared between who continued and dropped out of the study, by use of Chi-square tests.

As the emphasis in this study was on development of PTSD or PTSS post abortion, the responders from all three assessments were categorized into four groups [7], depending on their PTSD or PTSS trajectories: 1) Women who had *no* PTSD/PTSS at baseline but met the criteria for PTSD/PTSS at least once at the 3- or 6-month assessments were classified as having *developed PTSD or PTSS*. Women who had PTSS at baseline but met the criteria for PTSD at least once at the 3- or 6-month assessments were also included in this group, as these women transitioned from a subthreshold PTSD to PTSD during the study course. The rationale for this strategy was to fully evaluate the abortion as a potentially triggering experience. 2) Women who recovered, i.e., had PTSD or PTSS at baseline but no longer met the criteria for PTSD or PTSS, at the 6-month assessments were classified as *recovered.* 3) Women who met criteria for PTSD or PTSS at all assessments were classified as *unchanged*. 4) Women who never fulfilled criteria for PTSD or PTSS at any assessment were used as a *comparison group.*

The binary analyses of sociodemographic and clinical variables were analyzed with Chi2- tests. One-way ANOVA, followed by Tukey’s Honestly Significant Difference test, was used to compare personality trait scores between groups. These results were further subjected to a Bonferroni correction (*p*-value <0.004 for the personality scales and *p*-value <0.017 for the personality factors).

Risk factors for development of PTSD/PTSS post abortion was analyzed by multiple logistic regression analysis. Besides the Neuroticism factor, all available sociodemographic and clinical variables including abortion method and place for abortion, were considered as potential covariates, see Table 2. The final model included statistical significant variables in the bivariate analyses (*p* < 0.05) and variables based on the literature. Final covariates included age (dichotomized as <25 and ≥25, as most abortions in Sweden are performed in the age group 20 to 24 years followed by the age group 25 to 29 years [27, 28]. Educational level (< 12 years vs. ≥ 12 years, corresponding to completed high school or not), occupation (working full time, working part time, student, other occupation), experience of violence (yes or no), experience of sexual trauma (yes or no), alcohol use (yes or no), parity (yes or no) and counseling before the abortion (yes or no). In addition, previous abortion (yes or no) was included in the model, although it was not associated with PTSD development or Neuroticism in the bivariate analyses. However, prior studies have suggested that women with repeat abortions experience more adverse events, including violence, in their lives [36, 37], and would be expected to have higher levels of PTSD symptoms. Because of the close relationship between Neuroticism and mood disorders [16–19], anxiety and depression symptoms were not considered in the final analysis. The Neuroticism factor score was analyzed as a i) continuous variable, ii) categorized as above median vs. below median (reference) and iii) the highest quartile vs. below median (reference). SPSS Statistics for Windows, Version 20.0. (IBM Corp, Armonk, NY, USA) was used for all statistical analyses.

## Results

### Participants

Of the 1514 participating women at baseline, 13 were excluded because they chose not to have an abortion or had a second-trimester abortion. The SSP questionnaire was not completed by 122 women. Eighty-five women had not completed the entire SQ-PTSD questionnaire and could not be evaluated for presence of PTSD or PTSS; thus, 1294 women were available for analyses on Neuroticism-related personality traits in relation to PTSD or PTSS research diagnoses. Of the 1294 women who responded to the baseline questionnaire, 69 were never reported to the study center and consequently never received the follow-up questionnaire. Response rates were therefore 668/1225 (54.5%) at the 3-month follow-up, and 576/1225 (47%) at the 6-month assessment. Total numbers of responders over the two follow-up periods was 674, including six women who were responders at the 6-month assessment but were non-responders at the 3-month assessment.

Of the baseline responders, 92% were native Swedes. Foreign born women most commonly originated from Asia (3%), Europe (2%), other Nordic countries (1%), and South America (1%). Ages ranged from 15 to 52 years, with a mean age of 28 and a median age of 27 years (SD ± 7.2). Almost 70% had less than 12 years of education, and the most common occupational statuses were full-time work or student (Table 1).

**Table 1:** Characteristics and personality trait T-scores of responders and dropouts at the first visit at the clinic (baseline) and at follow-up after the abortion

	Baseline	3 month follow up			6 month follow up		
Variable	All (*n* = 1294)	Responders^a^ (*n* = 668)	Dropouts^a^ (*n* = 551)	*p-value*	Responders (*n* = 576)^b^	Dropouts (*n* = 98)	*p-value*
Age				<0.01			ns
15–19	115 (8.9)	49 (7.3)	58 (10.5)		41 (7.1)	8 (8.2)	
20–24	385 (29.8)	184 (27.3)	185 (33.6)		153 (26.6)	31 (31.6)	
25–34	516 (39.9)	277 (41.1)	209 (37.9)		239 (41.5)	38 (38.8)	
35–52	278 (21.5)	164 (24.3)	99 (18.0)		143 (24.8)	21 (21.4)	
Living alone	328 (26.2)	160 (24.3)	146 (27.8)	ns	133 (23.6)	27 (28.1)	ns
Education < 12 years	885 (68.9)	423 (63.0)	416 (76.3)	<0.001	352 (61.4)	71 (72.4)	<0.05
Occupation				ns			ns
Working full time	514 (41.0)	270 (41.1)	212 (40.0)		225 (40.1)	45 (46.9)	
Working part time	245 (19.5)	139 (21.2)	91 (17.2)		128 (22.8)	11 (11.5)	
Student	309 (24.6)	158 (24.0)	137 (25.8)		133 (23.7)	25 (26.0)	
Other occupation	187 (14.9)	90 (13.7)	90 (17.0)		75 (13.4)	15 (15.6)	
Anxiety symptoms	544 (42.4)	257 (38.4)	255 (46.8)	<0.01	217 (37.9)	40 (41.7)	ns
Depression symptoms	404 (31.5)	197 (29.4)	180 (33.0)	ns	167 (29.1)	30 (31.3)	ns
Antidepressant use	90 (7.3)	38 (5.8)	47 (9.2)	<0.05	33 (5.9)	5 (5.3)	ns
Alcohol use	1033 (80.1)	550 (81.6)	428 (78.2)	ns	466 (80.9)	84 (85.7)	ns
Tobacco use	499 (38.7)	222 (33.0)	254 (46.4)	<0.001	181 (31.5)	41 (41.8)	<0.05
Foreign born	104 (8.1)	41 (6.1)	49 (9.0)	ns	37 (6.4)	4 (4.1)	ns
No children	672 (56.0)	350 (54.8)	281 (56.1)	ns	301 (55.2)	49 (52.1)	ns
Previous abortion	448 (38.6)	208 (33.5)	211 (43.9)	<0.001	165 (31.3)	43 (46.2)	<0.01
Abortion method				ns			<0.05
Medical	1002 (81.9)	543 (83.4)	418 (82.1)		471 (84.7)	72 (75.8)	
Surgical	221 (18.1)	108 (16.6)	91 (17.9)		85 (15.3)	23 (24.2)	
Place of abortion				ns			ns
Home	645 (52.9)	360 (55.5)	274 (54.0)		310 (55.9)	50 (53.2)	
Clinic	574 (47.1)	289 (44.5)	233 (46.0)		245 (44.1)	44 (46.8)	
Counseling before abortion	464 (40.3)	280 (45.6)	181 (38.2)	<0.05	244 (46.6)	36 (40.0)	ns
Counseling after abortion	32 (2.8)	18 (2.9)	11 (2.3)	ns	17 (3.3)	1 (1.1)	ns
Neuroticism factor							
Somatic trait anxiety	51.8 ± 10.2	50.8 ± 9.9	52.7 ± 10.4	<0.004	50.5 ± 9.7	52.9 ± 11.0	ns
Psychic trait anxiety	49.6 ± 10.1	49.4 ± 10.1	49.8 ± 10.1	ns	49.5 ± 10.1	48.9 ± 10.1	ns
Stress susceptibility	52.8 ± 11.3	51.9 ± 11.2	53.7 ± 11.5	<0.004	51.8 ± 11.1	52.4 ± 11.6	ns
Lack of assertiveness	50.1 ± 10.0	50.6 ± 10.3	49.7 ± 9.9	ns	50.8 ± 10.2	49.2 ± 10.6	ns
Embitterment,	54.1 ± 12.2	53.1 ± 12.1	55.3 ± 12.3	<0.004	52.9 ± 12.2	54.0 ± 11.4	ns
Mistrust	51.8 ± 12.4	50.4 ± 12.0	53.5 ± 12.8	<0.004	50.4 ± 12.1	50.5 ± 11.7	ns
Aggressiveness factor							
Social desirability	49.5 ± 10.4	50.1 ± 10.1	49.2 ± 10.7	ns	50.2 ± 10.2	49.9 ± 9.4	ns
Trait irritability	52.0 ± 11.6	51.1 ± 11.6	52.5 ± 11.6	ns	50.8 ± 11.5	53.0 ± 11.9	ns
Verbal trait aggression	52.1 ± 10.5	51.1 ± 10.3	52.9 ± 10.3	<0.004	50.9 ± 10.3	52.4 ± 10.4	ns
Physical trait aggression	49.1 ± 11.1	47.8 ± 10.7	50.3 ± 11.3	<0.004	47.6 ± 10.7	49.3 ± 10.9	ns
Extraversion factor							
Impulsiveness	53.0 ± 10.2	52.4 ± 10.1	53.5 ± 10.3	ns	52.0 ± 9.9	55.1 ± 10.6	ns
Adventure seeking	52.5 ± 9.2	51.6 ± 9.2	53.3 ± 9.2	<0.004	51.2 ± 9.1	54.0 ± 8.9	ns
Detachment	48.1 ± 9.4	47.5 ± 9.3	48.7 ± 9.5	ns	47.6 ± 9.4	47.0 ± 8.8	ns
Neuroticism factor	310 ± 52	306 ± 52	315 ± 53	ns	306 ± 51	308 ± 54	ns
Aggressive factor	104 ± 34	100 ± 33	107 ± 35	<0.017	99 ± 33	105 ± 32	ns
Extraversion factor	57 ± 19	57 ± 19	58 ± 19	ns	56 ± 19	62 ± 19	<0.017

The bivariate analysis were performed by Chi2 test. Data are displayed in numbers (n) and percentages (%). Frequencies are given in relation to available information or responses. Missing information was prevalent in 0.1% (age) -13% (counselling after abortion). The personality trait scores were analysed by Independent t-test. Data are displayed in mean *(M*) and Standard Deviation (±SD)

^a^69 participants at baseline were not dropouts; they were never asked to participate at follow-up and therefore were excluded from the analyses

^b^Including 6 responders who were non-responders at the 3-month follow-up

*p < 0.004 for personality trait scores and *p* < 0.017 for personality factors, according to independent T-tests and Bonferroni correction

Because of the high number of dropouts in this cohort study an attrition analysis was conducted. The analysis showed that the dropouts to a higher extension were younger, had a lower level of education, were more often tobacco users, and had had a previous abortion more often than responders (Table 1).

### Sociodemographic data in relation to trajectories of PTSD or PTSS post abortion

Forty-eight women developed PTSD or PTSS after the abortion; of these 21 at some point developed PTSD and 27 developed PTSS. Among women who developed PTSS, eight fulfilled criteria for re-experiencing, avoidance and hyperarousal (B-D criteria), ten fulfilled at least two, and nine women fulfilled at least one of these criteria.

In addition, 137 women recovered from their PTSD/PTSS, and 25 women had an unchanged PTSD/PTSS status throughout the study course; while the majority (*n* = 464) had no PTSD or PTSS at any of the assessment points. Sociodemographic data in relation to the PTSD and PTSS trajectories are given in Table 2. In brief, women who at some point had suffered from PTSD or PTSS displayed higher baseline levels of anxiety and depressive symptoms and had more often experienced violence and/or sexual trauma than the comparison group. In addition, women who recovered and those who were unchanged in their status of PTSD/PTSS post abortion used more antidepressant medication than the comparison group. Women who developed PTSD/PTSS post abortion were to a greater extent young, had no children, had higher levels of anxiety and depressive symptoms, were more likely to be tobacco users but were drinking alcohol less often, and were in need of counselling before abortion more often compared with the comparison group (Table 2).

**Table 2:** Sociodemographic data and personality trait T-scores in relation to post-abortion PTSS/PTSD trajectories (*n* = 674)

Variable	Comparison group *n* = 464	Developed PTSD/PTSS *n* = 48		Recovered *n* = 137		Unchanged *n* = 25	
	n %	n %	*p-value*	n %	*p-value*	n %	*p-value*
Variable							
Age			<0.01		ns		ns
15–19	28 (6.0)	6 (12.5)		11 (8.0)		4 (16.0)	
20–24	119 (25.6)	22 (45.8)		39 (28.5)		4 (16.0)	
25–34	194 (41.8)	15 (31.3)		54 (39.4)		14 (56.0)	
35–52	123 (26.5)	5 (10.4)		33 (24.1)		3 (12)	
Living alone	108 (23.7)	16 (34.0)	ns	30 (22.7)	ns	6 (24.0)	ns
Education < 12 years	275 (59.5)	34 (72.3)	ns	95 (69.3)	<0.05	19 (76.0)	ns
Occupation			<0.05		ns		ns
Working full time	205 (45.3)	10 (22.2)		49 (36.6)		6 (24.0)	
Working part time	92 (20.3)	12 (26.7)		30 (22.4)		5 (20.0)	
Student	98 (21.6)	15 (33.3)		37 (27.6)		8 (32.0)	
Other occupation	58 (12.8)	8 (17.8)		18 (13.4)		6 (24.0)	
Sexual trauma	12 (2.6)	5 (10.4)	< 0.01	39 (28.5)	<0.001	9 (36.0)	<0.001
Experience of violence	79 (17.0)	19 (39.6)	<0.001	82 (59.9)	<0.001	18 (72.0)	<0.001
Anxiety symptoms	127 (27.5)	31 (64.6)	<0.001	80 (59.3)	<0.001	19 (76.0)	<0.001
Depression symptoms	107 (23.2)	18 (37.5)	<0.05	52 (38.5)	<0.001	20 (80.0)	<0.001
Antidepressant use	15 (3.3)	4 (9.3)	ns	16 (12.0)	<0.001	3 (12.0)	<0.05
Alcohol use	388 (83.6)	34 (70.8)	<0.05	110 (80.3)	ns	18 (72.0)	ns
Tobacco use	138 (29.8)	16 (33.3)	ns	52 (38.0)	ns	16 (64.0)	<0.001
Foreign born	25 (5.4)	3 (6.3)	ns	12 (8.8)	ns	1 (4.0)	ns
No children	239 (53.6)	29 (70.7)	<0.05	71 (55.5)	ns	11 (45.8)	ns
Previous abortion	135 (31.3)	14 (34.1)	ns	47 (38.2)	ns	12 (48.0)	ns
Abortion method			ns		ns		ns
Medical	377 (83.8)	35 (79.5)		110 (83.3)		21 (84.0)	
Surgical	73 (16.2)	9 (20.5)		22 (16.7)		4 (16.0)	
Place of abortion			ns		ns		ns
Home	257 (57.2)	22 (52.4)		68 (51.1)		13 (52.0)	
Clinic	192 (42.8)	20 (47.6)		65 (48.9)		12 (48.0)	
Counseling before abortion	175 (41.8)	25 (58.1)	<0.05	69 (53.9)	<0.05	11 (45.8)	ns
Counseling after abortion	10 (2.4)	3 (7.0)	ns	4 (3.1)	ns	1 (4.2)	ns

### Personality trait scores

Further attrition analyses by use of the pre-abortion personality assessments was performed (Table 1). Compared with responders, the dropouts at the three months assessment had slightly but significantly higher scores on Somatic Trait Anxiety, Stress Susceptibility Embitterment, Mistrust, Verbal and Physical Trait Aggression, and Adventure Seeking. There were no differences in personality traits between dropout and responders at the six-month assessment.

Personality trait scores in relation to the baseline, pre-abortion assessment of PTSD and PTSS are given in Table 3. As expected, women with PTSD or PTSS displayed higher scores than did the comparison group on almost all personality traits associated with Neuroticism, particularly Embitterment and Mistrust. The highest personality trait scores within the Neuroticism factor were found in women who fulfilled a research diagnosis of PTSD, whereas women with PTSS had intermediate scores, and the comparison group had scores close to the population mean (*T* score of 50). In addition, a number of personality traits associated with Aggressiveness and Extraversion was significantly higher in women with pre-abortion PTSD than in the comparison group (Table 3).

**Table 3:** Personality trait *T* scores of baseline responders on the Swedish universities Scales of Personality (*n* = 1294)

Variable	Comparison group *n* = 899	PTSD at baseline *n* = 89		PTSS at baseline *n* = 306	
	*M* ± SD	*M* ± SD	*p*-value	*M* ± SD	*p*-value
Neuroticism factor					
Somatic Trait Anxiety	49.6 ± 9.5	63.1 ± 9.4^a^	<0.001	54.8 ± 9.4	<0.001
Psychic Trait Anxiety	47.9 ± 9.7	58.8 ± 9.8 ^a^	<0.001	52.1 ± 9.4	<0.001
Stress Susceptibility	51.0 ± 10.7	62.4 ± 11.3 ^a^	<0.001	55.3 ± 11.1	<0.001
Lack of Assertiveness	49.5 ± 9.7	53.3 ± 11.5	0.002	50.9 ± 10.4	ns
Embitterment	51.1 ± 10.7	70.2 ± 12.7 ^a^	<0.001	58.4 ± 11.1	<0.001
Mistrust	49.4 ± 11.6	64.3 ± 11.5 ^a^	<0.001	55.1 ± 12.1	<0.001
Aggressiveness factor					
Social Desirability (negative loading)	49.9 ± 10.1	47.8 ± 12.5	ns	49.1 ± 10.5	ns
Trait Irritability	50.6 ± 11.4	58.6 ± 11.6 ^a^	<0.001	54.1 ± 10.9	<0.001
Verbal Trait Aggression	51.3 ± 10.3	56.1 ± 11.0	<0.001	53.4 ± 10.5	ns
Physical Trait Aggression	48.1 ± 10.6	53.8 ± 12.3	<0.001	50.8 ± 11.8	0.001
Extraversion factor					
Impulsiveness	51.9 ± 9.8	56.2 ± 11.6	<0.001	55.3 ± 10.4	<0.001
Adventure Seeking	52.1 ± 9.1	53.0 ± 10.9	ns	53.7 ± 9.1	ns
Detachment (negative loading)	47.6 ± 9.1	52.4 ± 9.9^a^	<0.001	48.5 ± 9.8	ns
Neuroticism factor	299 ± 48	372 ± 46^a^	<0.001	327 ± 47	<0.001
Aggressiveness factor	100 ± 33	121 ± 37	<0.001	109 ± 34	<0.001
Extraversion factor	56 ± 19	57 ± 23	ns	61 ± 20	ns

One-way ANOVA with post hoc Tukey HSD test. *M* = Mean; SD = Standard Deviation. The α-level was adjusted with Bonferroni correction; α = 0.004 for the individual personality traits and α = 0.017 for the personality factors. Unless otherwise stated, *p*-values are for comparisons with the comparison group

^a^ *p* < 0.001–0.002 in comparison with women who had PTSS at baseline

### Personality trait scores and PTSD or PTSS post abortion

Table 4 displays the baseline personality traits in relation to the PTSD and PTSS post abortion trajectories. Women who developed PTSD/PTSS post abortion had higher scores on Somatic Trait Anxiety, Psychic Trait Anxiety, Stress Susceptibility, and Embitterment (Table 4). Similarly, women who recovered and women who remained unchanged as to their PTSD/PTSS status throughout the study had higher scores than the comparison group on most of the neurotic personality traits. In fact, women with unchanged PTSD/PTSS status had the highest scores of Embitterment, and differed on this item from all other groups. Furthermore, women with recovered or unchanged PTSD/PTSS status also had higher scores on several of the personality traits within the Aggressiveness and Extraversion factors (Table 4).

**Table 4:** Personality trait T-scores in relation to post-abortion PTSS/PTSD trajectories (*n* = 674)

Variable	Comparison group (*n* = 464)	Developed PTSD/PTSS (*n* = 48)		Recovered (*n* = 137)		Unchanged (*n* = 25)	
	M ± SD	*M* ± SD	*p-*value	*M* ± SD	*p-*value	*M* ± SD	*p-*value
Neuroticism factor							
Somatic trait anxiety	48.7 ± 9.2	55.0 ± 9.6	<0.001	55.1 ± 10.0	<0.001	59.5 ± 9.6	<0.001
Psychic trait anxiety	47.4 ± 9.5	54.3 ± 8.5	<0.001	53.2 ± 10.5	<0.001	56.3 ± 9.4	<0.001
Stress susceptibility	49.6 ± 10.0	55.9 ± 11.0	<0.004	56.9 ± 12.6	<0.001	58.9 ± 11.0	<0.001
Lack of assertiveness	49.7 ± 9.6	54.6 ± 10.9	ns	55.5 ± 10.6	ns	51.1 ± 11.6	ns
Embitterment,	50.1 ± 10.4	56.6 ± 11.2	<0.001	59.2 ± 12.8	<0.001	68.5 ± 12.7^a,b^	<0.001
Mistrust	47.9 ± 11.0	53.7 ± 11.7	ns	55.7 ± 12.7	<0.001	61.5 ± 12.1	<0.001
Aggressiveness factor							
Social desirability	50.2 ± 9.7	51.4 ± 11.9	ns	49.5 ± 10.6	ns	50.3 ± 10.5	ns
Trait irritability	49.6 ± 11.4	53.3 ± 9.2	ns	54.5 ± 11.6	<0.001	56.3 ± 11.8	ns
Verbal trait aggression	50.1 ± 10.0	50.6 ± 9.1	ns	53.4 ± 11.1	ns	57.7 ± 10.5	<0.004
Physical trait aggression	46.5 ± 9.7	48.2 ± 11.3	ns	50.9 ± 12.4	<0.001	54.2 ± 11.6	<0.004
Extraversion factor							
Impulsiveness	51.1 ± 9.3	53.2 ± 12.2	ns	55.5 ± 10.6	<0.001	58.5 ± 11.1	<0.004
Adventure seeking	51.3 ± 8.8	51.4 ± 9.3	ns	51.8 ± 10.1	ns	57.6 ± 7.6	ns
Detachment	47.2 ± 8.8	50.0 ± 10.9	ns	47.7 ± 10.1	ns	48.3 ± 9.3	ns
Neuroticism factor	293 ± 46	330 ± 43	<0.001	332 ± 54	<0.001	356 ± 49	<0.001
Aggressive factor	96 ± 31	101 ± 32	ns	109 ± 36	<0.001	118 ± 34	<0.017
Extraversion factor	55 ± 18	55 ± 22	ns	60 ± 20	ns	68 ± 20	<0.017

One-way ANOVA with post hoc Tukey HSD test. *M* = Mean; SD = Standard Deviation. The α-level was adjusted with Bonferroni correction; α = 0.004 for the individual personality traits and α = 0.017 for the personality factors. Unless otherwise stated, *p*-values for comparisons with the comparison group are reported

^a^ *p* < 0.001 in comparison with women who developed PTSD/PTSS

^a^ *p* < 0.001 in comparison with women who recovered from PTSD/PTSS

High Neuroticism scores (continuous or categorized) and previous experience of violence were the strongest predictors for the development of PTSD/PTSS post abortion (Table 5). This finding was mainly driven by women who developed PTSD (*n* = 21), where the AOR for high Neuroticism scores and previous experience of violence were 6.73 (CI 95% 1.45–31.2) and 11.09 (CI 95% 3.00–41.0), respectively. Among women who developed PTSS post abortion, no significant predictors were found, data not shown.

**Table 5:** The association between age, neuroticism, alcohol use and counseling before abortion among women who developed PTSD/PTSS after the abortion (*n* = 48)

	Unadjusted		Adjusted for demographic variables	
Variable	OR	95% CI	OR	95% CI
Age	0.92	0.87–0.96	0.98	0.89–1.07
Education < 12 years	1.78	0.91–3.46	1.12	0.46–2.73
Occupation				
Working full time	1		1	
Working part time	2.67	1.12–6.41	2.31	0.80–6.65
Student	3.13	1.36–7.24	2.25	0.72–7.03
Other occupation	2.83	1.07–7.49	1.46	0.42–5.17
Sexual trauma	4.38	1.48–13.0	1.69	0.29–9.80
Experience of violence	3.19	1.71–5.98	2.72	1.21–6.12
Alcohol use	0.48	0.24–0.93	0.52	0.23–1.18
No children	2.09	1.04–4.21	1.32	0.42–4.13
Previous abortions				
0	1		1	
1	1.23	0.57–2.64	1.32	0.55–3.18
2	1.06	0.30–3.70	0.71	0.14–3.63
3 or more	0.73	0.09–5.75	0	n.c.
Counseling before abortion	1.94	1.03–3.66	1.20	0.56–2.56
Neuroticism				
Continuous	1.02	1.01–1.02	1.01^a^	1.003–1.02
High scores (above median vs. below median)	3.60	1.88–6.89	2.36 ^a^	1.08–5.16
Very high scores (highest quartile vs. below median)	4.81	2.26–10.22	3.22^a^	1.22–8.45

*OR* = odds ratio; *CI* = confidence interval

^a^adjusted for all variables in the table, except the other neuroticism variables

## Discussion

The most important finding was that women who developed PTSD or PTSS after the abortion scored higher on Neuroticism-related personality traits than did the comparison group who did not meet criteria for PTSD or PTSS prior to or after the abortion. Very high scores on the Neuroticism factor, together with experiences of violence, were associated with increased odds of developing PTSD or PTSS after the abortion. This finding was driven by the women who developed PTSD, whereas the risk factor profile for development of PTSS-only could not be determined.

As expected, women with PTSD at the pre-abortion assessment displayed higher scores than did the comparison group on almost all personality traits associated with Neuroticism, particularly Embitterment and Mistrust [13, 22]. Also, not surprisingly, the women who dropped out of the study scored higher on some of the Neuroticism-related personality traits than did women who responded to the follow-up questionnaires. This finding is in line with the dropout analyses in this cohort, which suggest that women with PTSD [7], and/or those with more pronounced anxiety symptoms at baseline are less likely to continue in this longitudinal study. However, although the high dropout rate of the study may have influenced the overall prevalence of PTSD or PTSS after the abortion [7], the influence of Neuroticism on PTSD or PTSS is likely unaffected by the drop-outs.

Our findings add understanding the reasons that some women develop mental health problems after an abortion by demonstrating that the individual level of Neuroticism-related personality traits may contribute to post abortion vulnerability to PTSD [13]. Among the many explanatory factors assessed, very high levels of Neuroticism emerged as the most influential factors for PTSD or PTSS post abortion. The findings is in accordance with previous studies indicating that high scores of Neuroticism is associated with PTSD [13, 16, 23] especially in women [22, 38]. However, contradictory results by Engelhart, et al. [39] demonstrate that pre-trauma Neuroticism was not significantly associated with PTSD symptoms after pregnancy loss. Instead, Engelhart and colleagues suggest that PTSD-arousal symptoms may overlap with Neuroticism [39].

Neuroticism is an important predisposing factor for major depression and anxiety disorders, including PTSD [16–24]. In the abortion context, this finding adds further support to the argument that the most consistent predictor of mental disorders after abortion is a pre-existing mental disorder [2–4]. Clearly, according to our results, not only pre-existing mental disorders but also personality features that may render a person susceptible to mental ill-health are of relevance. Extrapolating further to what is known in the field of depression and anxiety disorders, genetic factors shared with Neuroticism are commonly acknowledged to account for between one-third and one-half of the genetic risk of lifetime major depression, generalized anxiety disorder, panic disorder, and virtually all phobic disorders [17, 19, 40, 41]. Most well-known of these genetic factors is the short version of the serotonin transporter gene, which, among its many other effects, has been found to moderate the influence of stressful life events on depression [42]. Although less is known about the relationships between PTSD, Neuroticism, and shared genetic or environmental factors, we speculate that risk of post abortion mental health problems may also be influenced by genetic vulnerability traits [43] .

Moreover, exposure to violence was associated with increased odds of developing PTSD or PTSS after the abortion. This finding is in line with previous studies demonstrating that the highest risk of PTSD is associated with assaultive violence. This type of violence is more common among women than men [12], and seems more strongly associated with PTSD in females than in males [13].

An unexpected finding in our study, was that sexual trauma not was independently associated with development of PTSD post abortion. In contrast, population-based studies have suggested that sexual abuse is the traumatic exposure which most frequently lead to PTSD [13]. Intimate partner violence, where sexual violence is included, is more common among women seeking abortion than in other gynecology patients [36, 44], but Swedish women who request induced abortion are more likely to report exposure to physical violence than sexual violence [14, 36]. Potentially, sexual trauma is under-reported in our study, weakening the hypothesized association. Taken together, these findings stress the importance for health care professionals to approach women seeking abortion with questions of experiences of violence exposure.

Medical abortions in home settings are increasing in Sweden [27]. From a clinical point of view concerns has been raised that severe pain during the medical abortion may qualify as a traumatic experience. Clearly, neither the abortion method nor the place in which the abortion occurred contributed to this risk, and as previously shown by us, very few women who developed PTSD after the abortion did so because of trauma events that were related to the abortion per se [7]. Furthermore, similar results have been reported from studies of burn victims, where post-injury psychological health and PTSD were associated with baseline symptoms, avoidant coping, and Neuroticism-related personality traits, rather than severity of the burn [45, 46].

The personality trait that most prominently distinguished between patients who developed or remained unchanged in their PTSD status was Embitterment, which can be defined as persistent feelings of being insulted, a loser, or revengeful but helpless [47]. Although it is possible that women with PTSD or PTSS have reacted with embitterment to critical events in normal life [47], other explanations also include a greater degree of hostility among individuals with PTSD and PTSS [48] or self-blaming for not having been competent enough to avoid the trauma [47, 48] (feelings that may, in turn, lead to embitterment) [47]. Embitterment could also be considered to be associated with negative alterations in cognitions and mood that are associated with the trauma, which is described in DSM-5 as a new symptom cluster for PTSD. This D-cluster contains descriptions of negative beliefs or expectations about one self, others or the world, distorted blame of self or others and persistent negative emotional state [49]. Potentially, Embitterment captures some of the aspects of the new symptom cluster [39, 49].

Women who recovered or remained unchanged in their PTSD and PTSS status also had higher scores on some of the Aggressive personality traits. Anger and aggression are common symptoms in PTSD, among United States veterans as well as the general population [42, 48]. Previous study results indicate that the three PTSD symptom clusters of re-experience, avoidance, and hyperarousal from DSM-IV, may differently affect aggressive symptoms, where hyperarousal alone has been shown to be strongly associated with aggression [42, 50]. In DSM-5 the E- symptom cluster, alternations in arousal and reactivity that are associated with the traumatic event, with additional symptoms such as aggressive, reckless and self-destructive behavior has been taken to account [49]. So in addition, aggressiveness as well as embitterment might represent aspects where Neuroticism overlap with PTSD in the new DSM criteria [39]. Although additional knowledge for identifying persons at risk for post abortion PTSD might be gained by analyses of individual personality traits, PTSD symptom clusters, and the different ways posttraumatic distress may be expressed, the present study included too few women who actually developed PTSD following the abortion to allow such analyses. In addition, further research is also needed to define what interventions health care providers can offer to support women optimally.

As already mentioned, a major limitation of the study was the high dropout rate. Although women who appeared to be at risk for PTSD dropped out at a higher rate, this is unlikely to have influenced the overall relationship between Neuroticism and PTSD in the present study. Another limitation was the design of the questionnaire. The SSP inventory contains 91 items and was placed at the end of the questionnaire. As a consequence, some women did not complete the entire inventory, thereby further limiting the study sample at hand. Yet another limitation is that childhood trauma was not inventoried. Such measure would be relevant, as childhood trauma is related both to development of PTSD and Neuroticism [51, 52]. The homogeneity of the sample, consisting of a majority of Swedish natives is also a limitation of the generalizability of the study. Furthermore, the use of self- reported inventories has several limitations [53]. In the present study the research diagnosis of PTSD was based on self-reports from the inventory SQ-PTSD. The best way to evaluate a PTSD diagnosis is with the Clinician Administrated PTSD Scale (CAPS) [54], but this was not practical in the present study. However, the reliability and validity as well as the sensitivity and specificity of the SQ-PTSD have been tested with satisfactory results [11].

Notably, the study also has several strengths, such as the Swedish context where abortion has been accepted for almost half a century, and where the resistance to abortion is low and free of stigmatization compared to many other countries. The setting is somewhat ideal, free from confounders such as anti-abortion protesters, which may interfere and lead to negative experiences of the abortion.

## Conclusion

Findings in this study have strengthened the argument that the most consistent predictor of mental disorders after abortion is a pre-existing psychiatric disorder with which women present at the abortion clinic. We have shown that high scores on Neuroticism-related personality traits influence the risk of PTSD or PTSS post abortion. As expected exposure to violence also strongly influenced the risk of PTSD or PTSS after abortion

## Abbreviations

CI: Confidence Interval
DSM-5: The Diagnostic and Statistical Manual of Mental Disorders, Fifth edition
DSM-IV: The Diagnostic and Statistical Manual of Mental Disorders, Fourth edition
PTSD: Posttraumatic Stress Disorder
PTSS: Posttraumatic Stress Symptoms
SQ-PTSD: The Screen Questionnaire-Posttraumatic Stress Disorder
SSP: The Swedish universities Scales of Personality
